# Chinese families' knowledge, attitudes, and practices regarding seizure management for children with epilepsy: a mixed-methods study

**DOI:** 10.3389/fpubh.2023.1081720

**Published:** 2023-05-15

**Authors:** Cui Cui, Shuangzi Li, Wenjin Chen, Hengyu Zhou, Xianlan Zheng

**Affiliations:** ^1^National Clinical Research Center for Child Health and Disorders, Ministry of Education Key Laboratory of Child Development and Disorders, Department of Nursing Children's Hospital of Chongqing Medical University, Chongqing, China; ^2^National Clinical Research Center for Child Health and Disorders, Ministry of Education Key Laboratory of Child Development and Disorders, Neurology Department of Children's Hospital of Chongqing Medical University, Chongqing, China; ^3^School of Nursing, Chongqing Medical University, Chongqing, China

**Keywords:** children with epilepsy, knowledge-attitudes-practices, family, mixed-methods research, acute seizure

## Abstract

**Objective:**

This study aimed to examine Chinese families' knowledge, attitudes, and practices regarding the management of acute seizures (AS) that occur outside the hospital in children with epilepsy (CWE) and factors that influence AS.

**Design:**

A mixed-methods sequential explanatory study was conducted, which was integrated at the design and methods levels. In phase 1, a questionnaire was developed for this study, and a family functioning assessment was administered from Nov 2021 to Apr 2022. Multivariate logistic regression was used to analyze the knowledge, attitudes, and practices (KAP) and factors that influence AS. In phase 2, family caregivers (FCGs) were recruited from Jul to Aug 2022 to participate in a qualitative exploration, using semi-structured interviews and a combination of inductive and deductive methods.

**Setting:**

The setting was five children's specialty hospitals in different regions of China.

**Participants:**

The participants were FCGs of CWE. A total of 645 participants were included in the quantitative phase, and 15 FCGs (eight parents, five grandparents, and two others) were recruited for the qualitative phase.

**Results:**

The FCGs' average total KAP score for AS management was 66.23 ± 15.12, with 45.42% of FCGs having a low level. Univariate and multivariate regression analyses showed that demographic factors, disease characteristics, and family function significantly predicted family management of AS. The three most salient themes and eight sub-themes from phase 2 were explored. The quantitative and qualitative databases were analyzed separately and combined through integration, and a conceptual model was constructed based on the individual and family self-management theory (IFSMT); the model consisted of context, knowledge, self-regulation, and promotion factors.

**Conclusion:**

Chinese families have a positive attitude toward the management of out-of-hospital AS in CWE, but lack practice and related knowledge. AS management for CWE families was associated with the demographic characteristics of FCGs, epilepsy, and family characteristics. The research findings expand the existing application requirements of an Acute Seizure Action Plan and patient safety. Our results also indicate a pressing need for localized development of AS emergency medicine in family medicine, the establishment of auxiliary information systems, the utilization of caregivers' positive psychological resources, and improvements in family function for intergenerational care.

## Introduction

Epilepsy is one of the most common chronic neurological diseases in children, and ~60% of patients who have epilepsy have developed it in childhood. The incidence of epilepsy in Chinese children is 151 per 100,000, and about one-third of patients cannot control their seizures by taking drugs, resulting in Acute Seizures (AS) (1, 2). AS is the most common neurological emergency in children, including epileptic cluster seizures and status epilepticus, which may lead to death; the immediate complications of AS include metabolic acidosis and rhabdomyolysis, cognitive impairment, behavioral problems, and complex epilepsy. Proper family emergency interventions and care are crucial to improve the prognosis of patients with epilepsy (3). The unpredictability of AS seriously affects children's health and quality of life, and it has economic, psychological, and emotional effects on the entire family (4). Research results on children with AS show that it has a huge economic burden on families and the medical care system in terms of direct and indirect costs (5) and that the timely application of out-of-hospital emergency measures is negatively correlated with the number of hospital visits (6).

Research on the application of out-of-hospital AS management for children with epilepsy (CWE) shows that it is advantageous for patient safety (7) and reveals a positive developmental trend. The correct implementation of family AS first-aid for CWE improves the health status and the role of caregivers outside the hospital (8). An Acute Seizure Action Plan (ASAP) is an action guide for out-of-hospital epilepsy management that is provided by neuromedical professionals, such as patients, family members, school nurses, and other caregivers (9). The development and application of this tool emphasize the opinions and consensus of stakeholders (10). Families are the main caregivers of out-of-hospital seizures of CWE, and their knowledge, attitudes, and practices (KAP) and factors that influence them on out-of-hospital seizure management play a key role in the development and promotion of localized AS management tools (11, 12). Researchers in China and abroad have gradually paid increasing attention to the management of children's seizures that occur outside of hospitals, but there is still a lack of (1) understanding of the current situation and obstacles to the management of out-of-hospital AS in CWE from the perspective of families; (2) clarity about the social psychological theory of health behaviors that explain the possible factors that influence out-of-hospital AS management among CWE families; and (3) mixed-methods studies to comprehensively summarize these factors.

Therefore, this study aimed to understand the current situation regarding KAP related to AS management outside the hospital, using self-report questionnaires and semi-structured interviews with caregivers of CWE who were 0–18 years of age in China. An explanatory sequential mixed-methods design was used to explore the complex factors that influence prehospital seizure management to construct a conceptual model based on IFSMT.

## Methods

### Study design

The study used a mixed-methods sequential explanatory design and an inductive and deductive approach (13). In phase 1, 645 FCGs of CWE in China completed a questionnaire between Nov 2021 and Apr 2022, which was developed specifically for this study. Multivariate logistic regression was used to analyze the FCGs' KAP regarding AS and the factors that influence AS management. In phase 2, semi-structured interviews were conducted with 15 FCGs between Jul and Aug 2022 to collect qualitative data that were analyzed using a combination of inductive and deductive methods. The qualitative data from the perspective of the FCGs provided a greater depth of understanding of the quantitative data.

In addition to implementing integration at the design level, we also integrated at the methods level by connecting the qualitative phase to the quantitative phase (13), whereby the results from the quantitative phase informed the sampling criteria regarding the types of providers recruited for the qualitative phase. Furthermore, we implemented integration by building upon the quantitative data to refine our interview guide and develop our deductive codes.

**Figure 1:**
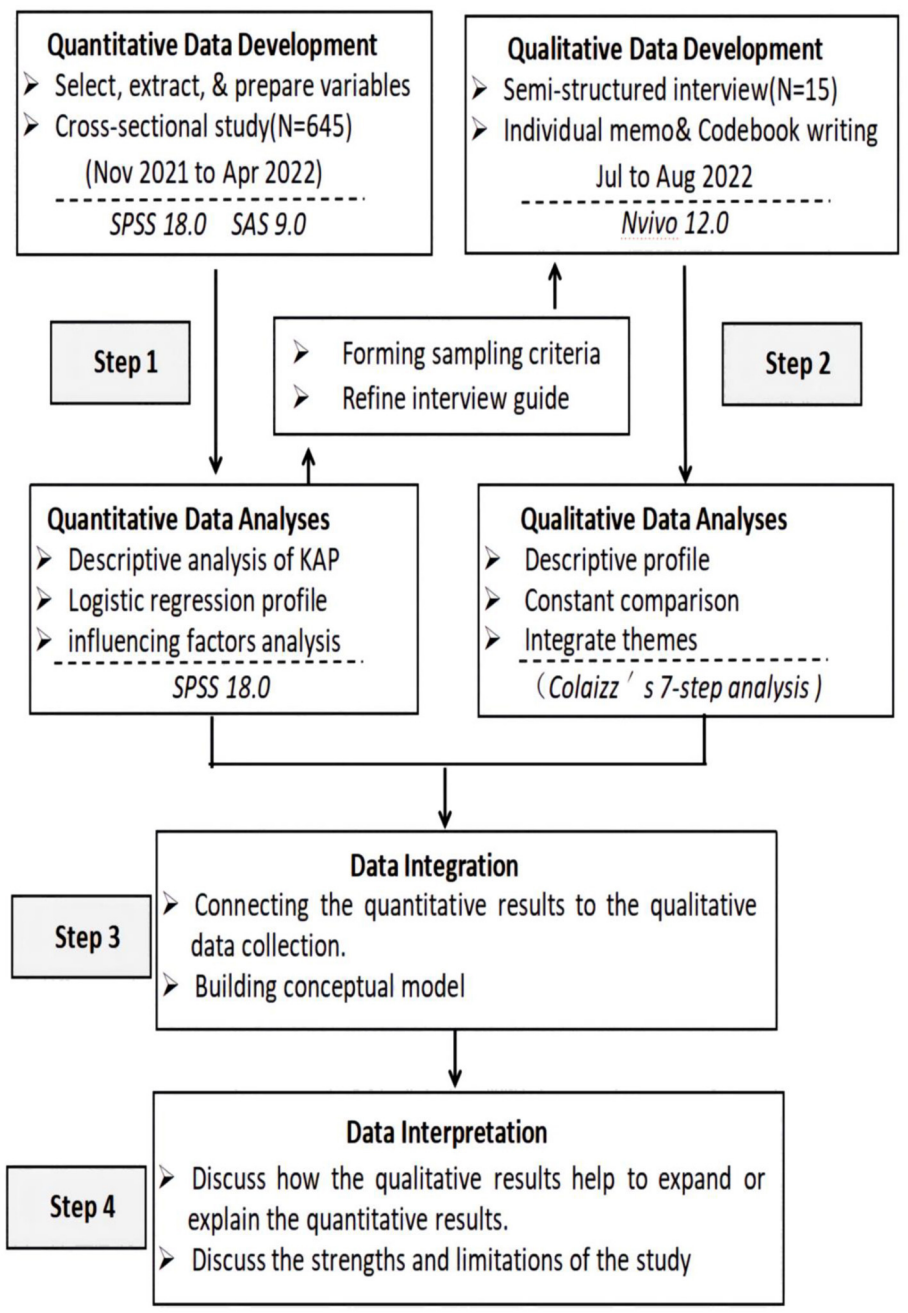
Algorithm for the mixed-methods expanded coverage design used in the study.

Figure 1 shows an overview of the study's methodology.

### Setting

This study was led by the Children's Hospital of Chongqing Medical University, one of China's top children's specialty hospitals. In 2021, the hospital registered 3,668,200 outpatient and 102,200 inpatient visits, with out-of-town patients accounting for nearly 40% of the total. Four other children's specialty hospitals in China were also included in the study, all of which are rated as guiding hospitals by the China Association Against Epilepsy; the admitted patients included regional coverage and are representative.

### Participants

#### Quantitative research

Convenience sampling was used to select the patient caregivers from the outpatients or inpatients of five departments of representative children's hospitals in different regions of China from Nov 2021 to Apr 2022. The inclusion criteria for CWE caregivers were as follows: (1) the family members lived with the CWE and took care of them for more than 8 h per day; (2) they had normal communication skills and were able to complete the questionnaire independently or with the help of the researcher; (3) they provided informed consent and voluntarily participated in this study; and (4) the children met the 2018 International League Against Epilepsy (ILAE) criteria for epilepsy seizures and classification, were 0–18 years of age, and had an AS within the past year. The exclusion criteria were FCGs who (1) had mental disorders or mental retardation or (2) recently suffered from serious physical diseases or serious accidents.

#### Qualitative research

The subsequent qualitative interviews were performed with volunteers who participated in the quantitative phase of the study. We initially used purposive sampling to identify family caregivers participating in roles that would provide unique insights into the contextual factors influencing the KAP of AS management patterns observed from our quantitative data. Initial recruitment targeted FCGs with different population and disease characteristics. Subsequently, we purposively sampled caregivers to explore AS-related topics that arose in the initial interviews further. We additionally used snowball sampling to identify other potential contributory roles, including patients with complex partial seizures or a history of status epilepticus seizures.

### Tools

#### Quantitative research

The questionnaire consisted of three parts.

##### General information questionnaire

This questionnaire was designed to collect demographic and other information about the participants.

##### Knowledge-attitude-practice questionnaire

The KAP was used to measure AS management of CWE who had AS that occurred outside the hospital. Relevant items in the self-made questionnaire were constructed in the framework of KAP theory. Relevant literature in China and abroad was searched (9, 11, 14–16). The questionnaire items were revised by eight experts from the National Pediatric Nursing Committee, the China Association Against Epilepsy, and experts on questionnaire design during three rounds of consultation; the expert authority coefficient was 0.96 (≥0.80). The final version of the KAP questionnaire for family prehospital AS management for CWE was composed of 34 items measuring three dimensions. These included 15 items about knowledge of acute episode management, 4 items on attitudes, and 15 items about practice during acute episode management. Each item was rated from 0 to 5, for a total score of 0–170. To facilitate comparison, the scores for each dimension and the total score of the TAP were converted into standard scores from 0 to 100: standard score = actual score/theoretical score **×**100. A standard score of >85 was considered excellent and a standard score of < 60 was considered poor. Standard scores between ≥60 and < 85 were considered good (17). The content validity index (CVI) of the KAP was 0.878, and Cronbach's α of the KAP for overall internal consistency was 0.783.

##### Family functioning assessment

The APGAR Index was developed by Dr. Smilkstein (18). This scale is an acronym for adaptation, partnership, growth, affection, and resolve, which is used to measure family support and help perform interventions to balance family relationships. The family function was classified as severe dysfunction (0–3), moderate dysfunction (4–6), and good family function (7–10). The Cronbach's α of the Chinese version was 0.817 (19).

#### Qualitative research

Semi-structured interviews were used to elicit data relating to individuals' perspectives on the prehospital management of AS. The prepared questions are shown in Supplementary Table 1. These questions were modified and combined (20) with the results derived from the quantitative part of this research to gain a greater understanding of the prehospital management of AS.

### Data collection

#### Quantitative research

The questionnaire was distributed to pediatric epilepsy specialist clinics and specialty wards of various medical institutions. All the researchers were trained specialized nurses with more than 15 years of work experience and good communication skills. The researchers distributed unified instructions, questionnaire completion instructions, and questionnaire access links to the survey; explained the study's purpose, significance, methodology, harmlessness, and anonymity to the participants; and obtained their informed consent prior to completing the questionnaire. Items that were not understood by FCGs were read and explained by the researchers, so the interpretation of the content of each item would be consistent. After the end of the survey, the validity and completeness of the data were checked. As the questionnaire contained four demographic variables, seven medical variables, and two scales (39 items), and we expected a 20% loss to follow-up, the study required a minimum sample size of 600 participants (21). Therefore, 712 questionnaires were distributed, of which 645 were returned, for a valid return rate of 90.59%.

#### Qualitative research

The semi-structured interview allowed participants to discuss each issue freely based on their own experiences. Interviews were conducted face-to-face by qualitative researchers. They were specialist nurses who had established trusted relationships with the children's parents through good communication and meticulous daily care. Following the principle of informed consent, they explained the purpose, methods, and content of the interview, promised to observe the principles of confidentiality and anonymity, and obtained the consent of the interviewees. The interviews lasted 30–35 min and were all digitally recorded and transcribed verbatim. Interviews were conducted until the thematic saturation was reached.

### Data analysis

#### Quantitative research

All the data were verified and checked by two authors and then imported into SPSS18.0 for statistical analysis. Frequencies, constituent ratios, and means ± standard deviations ($\bar{x}$ ± SD) were used for the descriptive analysis. The chi-square (χ^2^) test was used for comparisons, and multivariate logistic regression was used to analyze the predictor variables. The two-tailed level of statistical significance was α = 0.05.

#### Qualitative research

Transcripts were entered into Nvivo 12.0 software for storage and management, to ensure the integrity of the data analysis. We adopted a thematic analysis technique, which involved seven phases of data analysis (22): familiarization with the transcribed data, coding all relevant data, generating potential themes from the codes, making a thematic map, defining the themes' names, forming different topics, and a list of articles were returned to the participants to reach a consensus on the validity again. Themes were defined and named until the final findings were negotiated among team members. Two new participants were recruited to verify that saturation was reached. Their results contained all the important codes and topics that emerged from the previous participants, so it was decided that the sample size at this stage was sufficient (22).

## Results

### Quantitative results

#### General information and univariate analysis of KAP dimensions

The results show the demographic, medical, and family function information of all the participants. Table 1 shows the KAP scores' distribution regarding out-of-hospital AS management of FCGs and CWE by different characteristics.

**Table 1:** The distribution of the KAP scores of family caregivers and CWE, who conduct out-of-hospital AS management, by different characteristics.

**Variable**	**Items**	***N* = 645 (%)**	**KAP scores $\bar{x}$±*s***	***t/F***	***P***
**Demographic variables**					
Age of CWE (years)	< 3	67 (10.39)	58.38 ± 9.392	6.171	*P* < 0.01
	3–6	150 (23.26)	64.94 ± 17.374		
	7–13	335 (51.94)	62.22 ± 16.113		
	14–18	93 (14.42)	58.73 ± 17.517		
Course of disease (CWE)	< 6 months	93 (14.42)	57.41 ± 15.087	5.973	*P* < 0.01
	6–12 months	183 (28.37)	59.34 ± 15.842		
	13–23 months	186 (28.84)	61.61 ± 16.473		
	2–5 years	140 (21.71)	64.56 ± 16.076		
	>5 years	43 (6.67)	68.02 ± 20.146		
Education (FCGs)	High school or below	252 (39.07)	54.77 ± 11.485	69.060	*P* < 0.01
	Specialist	145 (22.48)	60.32 ± 14.522		
	Bachelor's degree or above	248 (38.45)	63.91 ± 18.909		
FCGs role	Mother/father	375 (58.14)	64.58 ± 17.203	32.721	*P* < 0.01
	Grandparents	237 (36.74)	55.41 ± 17.317		
	Others	33 (5.12)	46.11 ± 13.697		
Gender (FCGs)	Male	221 (34.26)	50.78 ± 15.341	0.823	0.331
	Female	424 (65.74)	53.35 ± 10.257		
Residence	District	310 (48.06)	56.28 ± 11.207	0.521	0.621
	Main urban	335 (51.94)	57.67 ± 11.561		
**Medical variables**					
Seizure type	Focal	311 (48.22)	58.88 ± 15.089	5.185	*P* < 0.01
	Generalized	184 (28.53)	67.69 ± 16.271		
	Mixed	88 (13.64)	63.23 ± 10.171		
	Others	62 (9.61)	57.05 ± 18.081		
Cause of epilepsy	Supernatural event	116 (17.98)	69.53 ± 19.514	4.132	*P* < 0.01
	Consanguineous marriage	53 (8.22)	56.34 ± 14.911		
	Brain damage	49 (7.6)	58.93 ± 16.367		
	Genetic	128 (19.84)	59.41 ± 14.764		
	Congenital	183 (28.37)	57.74 ± 14.209		
	Infectious	47 (7.29)	60.78 ± 17.004		
	Others	69 (10.7)	65.71 ± 13.107		
Primary signs of epilepsy (The most obvious symptom)	Postictal amnesia	46 (7.13)	50.72 ± 14.892	2.708	0.014
	Urinary incontinence	102 (15.81)	49.01 ± 13.529		
	Loss of consciousness	208 (32.25)	68.46 ± 16.843		
	Contractions	193 (29.92)	70.19 ± 17.137		
	Tongue bit	82 (12.71)	50.43 ± 16.242		
	Drooling	7 (1.09)	46.86 ± 17.460		
	Others	7 (1.09)	53.81 ± 21.996		
Seizure frequency	1 or more seizures daily	32 (4.96)	65.11 ± 9.327	30.015	*P* < 0.01
	1 or more seizures each week but not daily	123 (19.07)	67.03 ± 13.787		
	1 or more seizures each month but not weekly	22 (34.11)	68.42 ± 10.321		
	1–4 seizures each year	24 (37.52)	63.33 ± 11.005		
	Others	28 (4.34)			
Epilepsy management mobile application	In use	83 (12.87)	66.53 ± 16.047	6.573	*P* < 0.01
	No use	562 (87.13)	59.89 ± 16.346		
Antiepileptic drug	Monotherapy	233 (36.12)	56.23 ± 8.281	0.463	0.577
	Polytherapy	358 (55.50)	58.121 ± 10.121		
	Other	54 (8.37)	55.892 ± 12.630		
Family function	Severe dysfunction	211 (32.71)	55.85 ± 7.211	134.151	*P* < 0.01
	Moderate dysfunction	172 (26.67)	63.66 ± 11.798		
	Good function	262 (40.62)	68.68 ± 11.378		

#### Levels and scores on the KAP

The average standard total score of FCGs on the KAP was 66.23, and the mean standard scores for knowledge, attitudes, and practices are shown in Table 2. The items with the lowest scores were as follows: “timing of using emergency medication to relieve status epilepticus or recurrent seizures” (1.81 ± 1.05) on the knowledge dimension; “alleviate the severity of the exacerbation” (2.51 ± 1.46) on the attitude dimension; and “always carry information card about epilepsy disease signs” (1.23 ± 0.63) on the practice dimension. The items with the highest scores were as follows: “unable to be forcibly restrained during AS” (4.11 ± 0.32) on the knowledge dimension; “necessity of AS management action out-of-hospital” (4.35 ± 0.42) on the attitude dimension; and “when the child had frequent AS, bring her/him to the doctor in time or call 120” (3.78 ± 0.88) on the practice dimension.

**Table 2:** Levels and scores on the KAP in out-of-hospital seizure management for children (*N* = 645).

**Items**	**Excellent**	**Good**	**Poor**	**Scores $\bar{x}$ ±*s***
Knowledge	58 (8.99)	320 (49.61)	267 (41.40)	65.23 ± 13.39
Attitudes	106 (16.43)	507 (78.60)	32 (4.96)	76.15 ± 16.67
Practices	22 (3.41)	164 (25.43)	459 (71.16)	56.27 ± 15.55
Total	31 (4.80)	321 (49.77)	293 (45.42)	66.23 ± 15.12

#### Multivariate analysis of KAP dimensions

The variables that were significant in the univariate analyses were selected to be the independent variables (predictor variables) in the multivariate logistic regression, with the total KAP score as the dependent variable (Supplementary Table 2). Stepwise regression was used to screen meaningful variables (α_in_ = 0.10, α_out_ = 0.15). Caregiver role, educational level, course of the disease, type of epilepsy, presentation of epilepsy, application of mobile procedures, and family function were found to significantly predict total KAP scores, as shown in Table 3.

**Table 3:** Multivariate analysis of KAP in out-of-hospital AS management for children.

**Items**	**Coefficient**	**Standard deviation**	***T***	***P***	**[95% Conf. Interval]**	
FCG role	−1.502	0.699	−2.150	0.032	−2.875	−0.128
Education (FCGs)	3.069	0.479	6.410	0.000	2.129	4.009
Course of disease (CWE)	0.762	0.346	2.200	0.028	0.082	1.442
Seizure type	−3.517	0.917	−3.840	0.000	−5.317	−1.717
Primary signs of epilepsy	−0.319	0.336	−0.950	0.044	−0.979	0.342
Epilepsy management mobile application	−0.642	0.994	−0.650	0.019	−2.594	1.311
Family function	−5.997	0.486	−12.340	0.000	−6.951	−5.043
Constant	67.201	3.443	19.520	0.000	60.439	73.962
Age (CWE)	−0.560	0.457	−1.220	0.221	−1.458	0.338
Cause of epilepsy	−0.245	0.323	−1.247	0.058	−1.583	2.673

### Qualitative results

Among various topics discussed during the interviews, the three most salient themes of factors that influenced KAP in AS management for CWE out-of-hospitals were recognized, and eight subthemes also emerged from the analysis. The qualitative results are as follows. **Theme 1:** health promotion needs for AS management outside the hospital; **Theme 2:** knowledge factors (three subthemes, including information reservation, personal characteristics, and decision-making ability); **Theme 3:** Attitude factors (two subthemes, including positive psychology and risk perception); and **Theme 4:** Practice factors (three subthemes, including resource availability, support and share, and effective training). The thematic analysis of qualitative results and excerpts of representative interview segments are shown in Supplementary Table 3.

### Merging of the qualitative and quantitative data

We found that the results of the two phases complemented each other based on the mix-method research (13), including information reservation, personal characteristics, share support, and risk perception. The interview content deepened the descriptive statistics of the KAP and the regression results of factors predicting the AS management for CWE outside the hospital. (1) In the quantitative study, the score on the knowledge dimension was 65.23, which was at a medium to low level. In the interview, participants found that there were obstacles to their knowledge acquisition, understanding, and application of AS management of the theme information reservation. (2) In the quantitative study, grandparents accounted for 36.74% of FCGs, and their KAP score was 55.41, which was low; therefore, grandparents were included in the qualitative study to explore the personal characteristics of AS management. (3) In addition, multivariate regression results indicated that family function predicted KAP in AS management (*t* = −12.340, *P* < 0.001). The interviews found that the company, support, and joint participation of family members provided support for the good experience and motivation of family caregivers involved in sharing support. (4) Recognition of the main symptoms of epileptic seizures also predicted KAP (*t* = −0.950, *P* = 0.044). The cognitive level of AS management for alleviating the severity of seizures had the lowest mean on the attitudes dimension (2.51 ± 1.46). The perceived risk of AS in caregiver involvement was the qualitative encoding in AS management, for which explanation was evident.

Moreover, some other factors had a supplemental influence, as revealed in interviews. (1) AS and epilepsy-related knowledge of caregivers was low (65.23). Characteristics of epilepsy disease (course, type, and manifestations) and the educational level of caregivers were also included in the regression model. In the interview, participants indicated that the obvious obstacles were uncertainty about the timing and content decisions of AS management, which we merged into subthemes of decision support. (2) Disease course was also found to be a predictive factor (*t* = 2.200, *P* = 0.028). The longer the duration of the disease course, the higher the KAP score was, thus caregivers of CWE who had a disease course >5 years were selected for an interview. The caregivers' resistance and incomprehension at the initial stage of the disease changed with the increase in the disease course. We expanded the theme of positive psychology. (3) In addition, participants expressed expectations for effective resources, including convenient follow-up information platforms, wearable seizure tracking devices, AS location, and other software. The results of epilepsy management mobile applications in the quantitative research were expanded (*t* = −0.650, *P* = 0.019), which was listed as an auxiliary resource. (4) Finally, the score on the practice dimension was 56.27, which was low. In the interview, participants expressed the lack of a professional, standardized, simple, and easy-to-follow guidance list or tools for AS management, which was expanded to the theme of effective tools.

**Figure 2:**
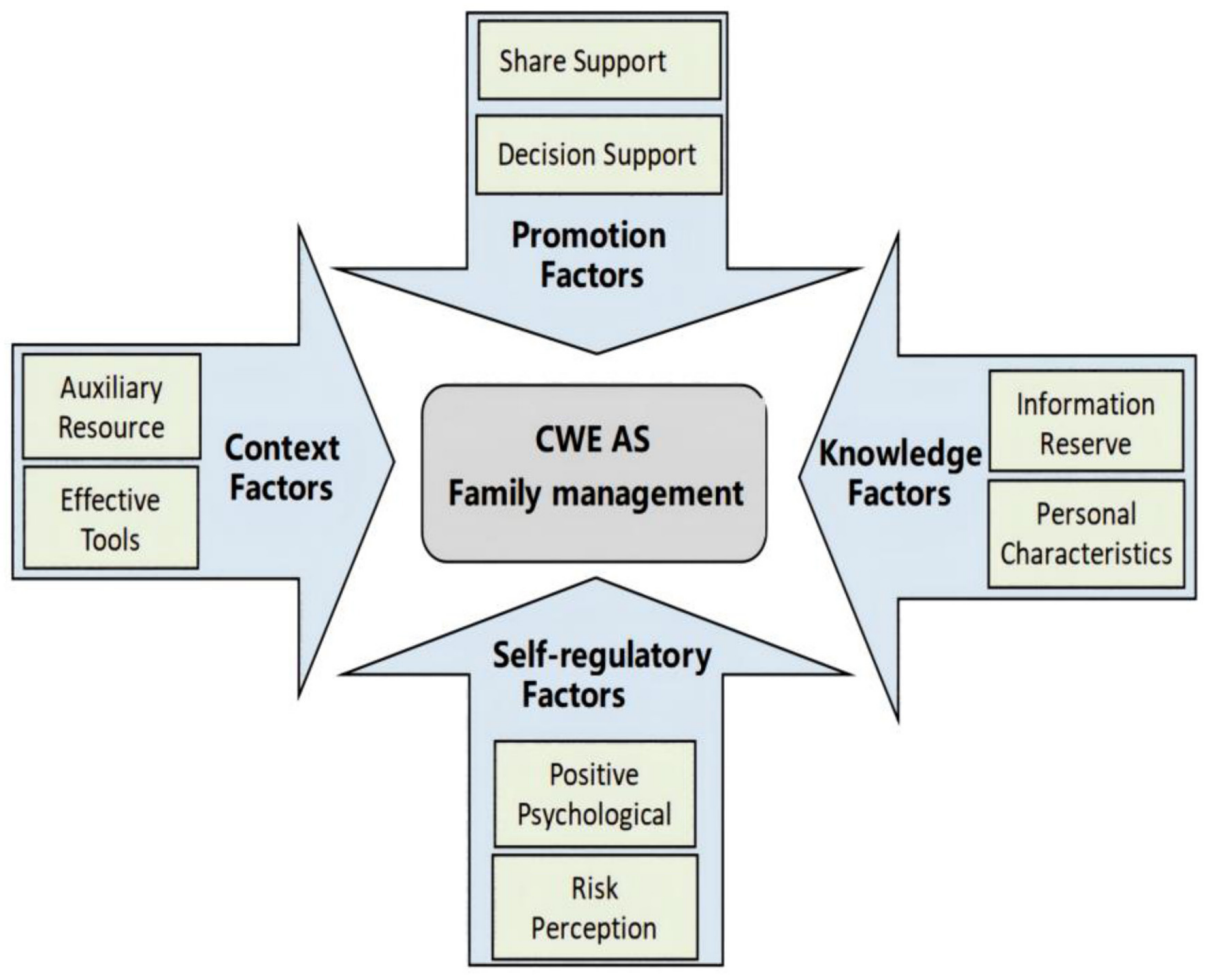
Conceptual model of the factors that influence AS family management.

Therefore, we created a loose conceptual model based on the KAP model and the individual and family self-management theory (IFSMT) to classify and visualize the set of factors that were found. Since AS management was the main concern of this study, it was selected as the central action, which linked the main results (Figure 2). (1) The first was context factors (i.e., auxiliary resources and effective tools), which were included in the situation dimension of IFSMT, affecting the specific conditions and objective resources of family caregivers' self-health management. (2) The second was knowledge factors, including information resources and personal characteristics, which reflect the knowledge and belief elements of the IFSMT process dimension and refer to the accumulation and application of information affecting the AS management outside the hospital in the epilepsy family. (3) The third was promotion factors, that is, sharing and decision support, which belong to the guiding and coordinating ability elements of the IFSMT process dimension and refer to social support and its influence on AS management, as well as comprehensive judgment and execution ability. (4) The fourth was self-regulating factors, including positive psychology and risk perception that reflect the self-regulating skills of the IFSMT process dimension, which refers to the self-emotional and cognitive requirements in AS management.

## Discussion

### Status of KAP regarding seizure management for CWE

The results showed that the current situation of knowledge, attitudes, and practices related to managing out-of-hospital AS for CWE is not optimistic in China. In this survey, the KAP score of AS management for CWE was 66.23 of which 45.42% of FCGs were at the poor level. Surveys about epilepsy disease and treatment of CWE in different countries have also revealed misconceptions and negative attitudes among parents (23, 24). (1) In the knowledge dimension, “not knowing when to use emergency medication to relieve status epilepticus and recurrent seizures” had the lowest score. The interviews found that the choice of first-aid drugs was limited, and it was difficult to judge the application time and method, mainly due to the limited development of appropriate dosage forms of first-aid drugs, the low frequency of first-aid drug prescriptions, and insufficient training. It is suggested that the drug management environment and policies in specific areas should be combined, and it is necessary to consider the characteristics and acceptability of drug use in children of different ages and promote the development, approval, and training of appropriate dosage forms for AS first-aid drugs. (2) In the attitude dimension, the cognitive score for “out-of-hospital seizure management will alleviate the severity of seizures” was the lowest. The caregivers expressed in the interview that only taking medication on time and follow-up were directly related to the frequency of children's seizures, which suggests that medical staff should adjust the content of health education for families who participate in AS management. (3) In the practice dimension, the lowest score was for “A sign for your child to carry information about epilepsy.” The reason is that signs create stigma for children and families, according to the interviewed FCGs. Although the strength of “stigma” associated with epilepsy may be related to internal factors (such as low self-esteem), external factors also exist, including cultural differences (25). It is suggested that it is also important to conduct social and family acceptance education for epilepsy in combination with cultural and individual differences when promoting an accurate understanding of epilepsy in society, as a whole.

### Model of the factors that influence AS family management

The mixed-methods study facilitated contact between healthcare workers and families who have CWE, improved insight into the current status of the KAP management for CWE, and it also deepened the situational understanding of AS management. The results showed that the factors affecting out-of-hospital AS management in Chinese families with CWE are complex and systematic. This conceptual model provides a reference point for developing AS management training programs for families, improving management intervention programs, designing localized ASAP tools for children, and developing effective auxiliary information systems.

#### Context factors

A single out-of-hospital AS training session for CWE is not suitable for all epileptic children and their families (11). Individualized, focused, dynamically assisted, and succinct AS management tools are the key to solving CWE family problems. This study found that the practice scope of family AS management for children has expanded and that the demand for it is obvious. By reviewing out-of-hospital AS records and follow-up medical records of CWE, researchers can further understand the occurrence of AS and its developmental process. This research will also help to promote a family AS management plan for local CWE based on “big data.” A clinical translational study should be conducted on CWE of different ages, who have different family structures and who live in different regions to verify the value of family AS management for children and its effectiveness in improving the quality of medical care and reducing medical costs.

At the same time, the impact of epilepsy management software on KAP indicates that a combination of intelligence and AS management is needed to break through the traditional disease follow-up model. The combination of seizure monitoring, medication management, health education, and disease follow-up through Internet technology can improve the accessibility of family AS management to a certain extent. Moreover, the government should strongly support the implementation of research on prehospital AS management, establish hospital-family-society first-aid information sharing and links, and strengthen collaborative services among hospitals at all levels to finally achieve a reduction in AS and the adverse consequences of seizures.

#### Knowledge factors

Knowledge inculcation and belief building play a supporting role in individual behavioral change (26). This study suggests that CWE families need to establish a correct and complete understanding of AS. According to the characteristics of CWE families, the information resource should have the following characteristics: (1) Convenient access to information about the characteristics of AS, which is not restricted by time, place, or conditions, so that information is readily available; (2) Scientific content, in which educational information for CWE families is summarized based on high-quality evidence about taking care of CWE at different ages and disease states, for families who live in different areas and have different experiences; (3) Information acceptability because most caregivers have non-medical backgrounds and need an illustrated or easy-to-understand educational checklist; and group demonstrations and peer education should also be included.

As personal characteristics also affect caregivers' utilization of AS knowledge, intergenerational parenting is obviously a solution to China's aging problem and the increasing cost of childcare. This study suggests that grandparents cannot compensate for the absent parents of CWE to provide family AS management. We are encouraging more young parents to participate in the daily care of children with chronic diseases. Meanwhile, developing management tools and apps suitable for intergenerational parenting are helpful strategies for improving AS management in local CWE families.

In addition, caregivers' adverse experiences can stimulate their confidence in family AS management. Protection motivation theory believes that information resources and previous behavioral experience can stimulate cognitive assessment (27). Caregivers who have experienced serious illness or whose family members suffer from chronic diseases may have a deeper understanding of the disease and be more inclined to participate in health management (28). Family-centered assessments of personal characteristics and interventions of caregivers by medical staff will help patients and FCGs to embrace the knowledge of AS management.

#### Promotion factors

Based on our IFSMT analysis of the factors that influence AS management in CWE families, support should include sharing support within the family and between the family and the external environment. This study shows that family function is a factor that influences the KAP status of AS management. The interviews revealed that better relationships among family members can contribute to AS management practices. Urgent attention should be given to determining how to optimize the internal resources of families and stimulate the internal motivation to deal with AS management. In addition to the social acceptance of epilepsy, family AS management requires financial, material, human, policy, and leadership support. Therefore, it is necessary to explore the cooperation among pediatric neurological specialists and community and family caregivers to develop local ASAP training programs. Strengthening the authorized management of prehospital first-aid drugs for community nurses is also needed, as well as improving family-community-hospital green emergency channels.

The subtheme of decision support involves FCGs being able to recognize seizure symptoms correctly and the timely use of first-aid medications. However, due to the lack of out-of-hospital emergency drug utilization among local CWE (29), combined with the safety, effectiveness, and convenience of anticonvulsant use in children, it is urgent to develop single-dose fixed dosage forms or non-invasive dosage forms. Medical institutions need to strengthen the support of health education about rational drug use, including multiple forms of AS drug training for family caregivers and school staff, such as AS drug use methods, medication timing, and observation of adverse drug reactions.

#### Self-regulatory factors

The International League Against Epilepsy Psychology Task Force stated that mindfulness treatment that emphasized coping with stress was the highest evidence-based intervention for psychological support for children's self-management of epilepsy (30). Therefore, it is necessary to consider incorporating mindfulness training into behavioral training or adjuvant therapy for family AS management, which should be conducive to overcoming family cognitive dysfunction and stimulating positive psychological feelings. In addition, this study attempted to improve caregivers' perception of the harm of bad AS management behavior and the benefits of healthy behavior through patient safety education, to promote the positive adoption of healthy behavior.

## Conclusion

This study used a mixed-methods approach to explore the complex factors that affect the knowledge, attitudes, and practices of Chinese families regarding out-of-hospital AS management for CWE and constructed a conceptual model that included context, knowledge, promotion, and self-regulatory factors based on the IFSMT and KAP framework. Self-management of CWE under medical care prioritizes “the voice of the patient” and “the positive role of the family in their own health care,” leading to more responsive services and better care outcomes (30). However, improving the KPA of family caregivers of CWE cannot replace the role of professionals. Therefore, research is needed to determine how to relate, compliment, and balance the relationship between them, and how to evaluate the availability and effectiveness of out-of-hospital AS management for children. This will require the development of objective outcome indicators for various interest groups. This study offers a novel framework to guide ASAP existing knowledge that has the potential to generate unique insights into multifaceted phenomena related to usability.

## Limitations

This study has three main limitations, which can enlighten future research. First, the conceptual model of AS management and the four factors that affect it need to be verified statistically. Second, the present study used a cross-sectional design; thus, it was unable to explain changes in the KAP of AS management for CWE families from childhood to adulthood. Finally, research on the interaction among stakeholders and healthcare providers' perspectives on AS management behavior needs to be explored.

## Data availability statement

The raw data supporting the conclusions of this article will be made available by the authors, without undue reservation.

## Ethics statement

The studies involving human participants were reviewed and approved by Children's Hospital of Chongqing Medical University. All of the participants provided oral consent and expressed their willingness to complete the study, and written informed consent was obtained from 15 FCGs as interviewees.

## Author contributions

XZ and CC contributed to the conception and design of the study. CC contributed to the writing of the research proposal that was submitted for ethical consideration, developed the qualitative investigation and analysis, and prepared the manuscript for submission to the journal. SL and WC collected both quantitative and qualitative data, extracted and transcribed the data, and performed the data analysis. HZ contributed to the recruitment of the subjects. All the authors have read and approved the published version of the manuscript.
