# Supplementary material for: Chinese families' knowledge, attitudes, and practices regarding seizure management for children with epilepsy: a mixed-methods study
Source: Front Public Health. 2023 May 15;11:1081720. doi: 10.3389/fpubh.2023.1081720 (PMC10225546; doi:10.3389/fpubh.2023.1081720)
Supplement: Supplementary file 1 [file Data_Sheet_1.docx]

**Chinese Families’ Knowledge, Attitudes and Practices Regarding Seizure Management for Children with Epilepsy: A Mixed- Methods Study**

**Supplementary Material**

**Supplementary Table 1. Semi-structured interview guide**

|  |
| --- |
| 1. As a family caregiver, what do you think are the emergency situations for children with seizures? |
| 2. What difficulties do you have in acquiring and understanding the knowledge of acute seizures management in children with epilepsy outside the hospital? |
| 3. As a family caregiver, what do you think of the acute seizures management for children with epilepsy outside the hospital? |
| 4. What difficulties do you have in dealing with acute seizures for your child outside the hospital? What are the influencing factors? |
| 5. How do you feel and experience when caring for children with acute seizures outside the hospital? |
| 6. What help and support do you think family caregivers need in acute seizure management out of the hospital? |

**Supplementary Table 2. Independent variable coding assignments**

| **Independent variable** | **Coding Assignment** |
| --- | --- |
| Age (CWE) | Set dummy variable with <3 years as reference |
| Education (Participants) | Set dummy variable with reference to high school and below,  range of assignment: junior college, university and above |
| FCGs role | Set dummy variable with reference to parent, range of assignment: grandparent , other |
| Seizure type | Set dummy variable with local seizure as reference, range of assignment: generalized seizure , other syndromes |
| Cause of epilepsy | Set dummy variable with trauma events as reference, range of assignment: close relative marriage, brain injury, genetic , congenital, infectious, loss of consciousness |
| Number of signs of epilepsy | Numerical variable |
| Epilepsy management mobile application | Set dummy variable with in use as reference, range of assignment: use, useless |
| [Course of disease](file:///C:\\Users\\工作\\20220914%20数据-问卷分析-国内-1500\\javascript:;) | Numerical variable |
| Family function | The dummy variable was set with severe family dysfunction as the reference, range of assignment: moderate family dysfunction , good family function |

**Supplementary Table 3. The thematic analysis of the qualitative results and excerpts of representative interview segments**

| **Theme 1: Health promotion needs for AS management outside the hospital** | | |
| --- | --- | --- |
| **Main thematic analysis** | | **Interview segments** |
| Most participants exhibited a positive attitude and demand for out-of-hospital AS management, and believed that the uncertainty and repeatability of acute episodes were troubling to the lives of children and their families, and they admitted that correct and timely out-of-hospital AS management could alleviate family and social pressure. At the same time, the caregivers said that learning about out-of-hospital seizure management and participating in the treatment of children's seizures would help them have a sense of control over the disease. | | *"My nerves have been on edge, I am afraid of his sudden seizures and accidental injury, and I hope that the hospital's attention to patients can extend to the family." P3*  *"It's great that the website provided by the hospital has videos of first-aid for AS, which can* *provide a reference for us to deal with children's seizures at home." P1* |
| **Theme 2: Knowledge factors** | | |
| **Subtheme** | **Main thematic analysis** | **Interview segments** |
| information reservation | Especially in the early stages of the disease, most FCG often felt overwhelmed. The lack of disease knowledge also made FGCs less likely to be vigilant in monitoring abnormal symptoms of their children before an AS, leading to a failure to provide timely emergency interventions for children. | *"The nurse said to avoid tongue bites during the seizure, you can put in hard objects. One time I put chopsticks in the child's mouth and made a gap between the two front teeth. " P6*  *"I don't know where to look for specific health guidance, especially with our kind of complicated epilepsy, which has a lot of special problems." P10* |
| personal characteristics | The family management of AS was influenced by intergenerational care in the traditional culture. Because grandparents may have lacked updating knowledge and ability, and energy, the uncertainty of first-aid AS management out-of-hospital was obvious.  In addition, caregivers who had experience with self-management of an illness themselves expressed their confidence in their out-of-hospital AS management of their children. | *"His mother gives him to me before she goes to work outside. There was no problem in taking care of him, but I was old and had a poor memory. He sometimes had seizures at home, and I could only worry." P12*  *"I have diabetes, and I know the significance of self-care for patients with chronic diseases. I am confident that I can help my children overcome the problems caused by AS " P11.*  *"I'm very nervous that if I can't do it well, I will not relieve the seizure, but increase the damage to the child." P7* |
| decision-  making ability | Some caregivers could timely assess the predisposing factors or characteristics of an AS attack based on daily observation and knowledge accumulation, which was conducive to their timely participation in AS management. | *"Her behavior is not typical (absence seizure). Once I find that she is staring blankly or standing frozen and does not respond to a call, I will immediately hold her up, because after 3-5 seconds, she will generally have lower limb weakness and fall suddenly." P11* |
| **Theme 3: Attitude factors** | | |
| **Subtheme** | **Main thematic analysis** | **Interview segments** |
| positive psychology | Participants reported that they needed warm, encouraging, and vivid experiences or examples to motivate them to face the challenges and difficulties of out-of-hospital AS management. | *"I keep reminding myself and my children that AS is part of my family and we need to accept it (AS). In this way, we'll be more relaxed." P13* |
| risk perception | Caregivers perceived the benefits of health management and the disadvantages of risky behaviors, which helped them perform out-of-hospital AS management. | *"I observed that staying up late and playing video games for a long time would induce his (CWE) AS. After that, when we regulated his bedtime and limited his gaming time, the seizures did decrease." P9* |
| **Theme 4: Practice factors** | | |
| **Subtheme** | **Main thematic analysis** | **Interview segments** |
| resource availability | Most of the caregivers said they used the Internet to find out about AS management outside the hospital. They admitted that these websites lacked expertise and authority, and sometimes even provided wrong answers. In the interview, only one caregiver mentioned that he would supplement emergency management measures by consulting professional books. | *"The child had frequent seizures at home, and we recorded a whole video because the children's hospital was far away from home, unable to see patients on site, and the Internet inquiry could not upload all the videos. I think the doctor did not know the child's condition." P8* |
| support and share | In the process of out-of-hospital AS management, family caregivers paid more attention to support from others.  Some caregivers passed on support to other families by sharing knowledge and care experiences. | *"My parents give me great support, they help me take care of my other child and let me pay more attention to him (CWE)." P10*  *"I didn't want new patients to make the same mistakes, so I opened an official account, and provided AS management experience." P4* |
| effective training | Caregivers reported disparities in out-of-hospital practical and theoretical learning, and one health education package was limited for each child due to differences in age, seizure subtype, seizure type, and family environment. | *"At the hospital, the nurse gave us a first aid demonstration, but I still didn't know what to do when the child has a seizure at home." P5*  *"The education leaflet tells us that if the seizure lasts for five minutes and it is not relieved, we should send him to the nearest hospital for treatment. We live in the countryside, and it takes nearly half an hour to drive to the nearest hospital. What should we do?" P8* |
